# Chicken interferome: avian interferon-stimulated genes identified by microarray and RNA-seq of primary chick embryo fibroblasts treated with a chicken type I interferon (IFN-α)

**DOI:** 10.1186/s13567-016-0363-8

**Published:** 2016-08-05

**Authors:** Efstathios S. Giotis, Rebecca C. Robey, Natalie G. Skinner, Christopher D. Tomlinson, Stephen Goodbourn, Michael A. Skinner

**Affiliations:** 1 Section of Virology, Faculty of Medicine, Imperial College London, St. Mary’s Campus, Norfolk Place, London, W2 1PG UK; 2 Faculty of Medicine, Imperial College London, London, SW7 2AZ UK; 3 Bioinformatics Support Service, Imperial College London, Sir Alexander Fleming Bldg, London, SW7 2AZ UK; 4 Institute for Infection and Immunity, St. George’s, University of London, London, SW17 0RE UK

## Abstract

Viruses that infect birds pose major threats—to the global supply of chicken, the major, universally-acceptable meat, and as zoonotic agents (e.g. avian influenza viruses H5N1 and H7N9). Controlling these viruses in birds as well as understanding their emergence into, and transmission amongst, humans will require considerable ingenuity and understanding of how different species defend themselves. The type I interferon-coordinated response constitutes the major antiviral innate defence. Although interferon was discovered in chicken cells, details of the response, particularly the identity of hundreds of stimulated genes, are far better described in mammals. Viruses induce interferon-stimulated genes but they also regulate the expression of many hundreds of cellular metabolic and structural genes to facilitate their replication. This study focusses on the potentially anti-viral genes by identifying those induced just by interferon in primary chick embryo fibroblasts. Three transcriptomic technologies were exploited: RNA-seq, a classical 3′-biased chicken microarray and a high density, “sense target”, whole transcriptome chicken microarray, with each recognising 120–150 regulated genes (curated for duplication and incorrect assignment of some microarray probesets). Overall, the results are considered robust because 128 of the compiled, curated list of 193 regulated genes were detected by two, or more, of the technologies.

**Electronic supplementary material:**

The online version of this article (doi:10.1186/s13567-016-0363-8) contains supplementary material, which is available to authorized users.

## Introduction

The interferon (IFN) response is one of the most important arms of host innate immunity against virus infection [1, 2]. Infected cells are able to recognise foreign nucleic acids and induce the synthesis and secretion of type I IFN (IFN-α and IFN-β) and type III IFN (IFN-λ), which bind to receptors on the surface of neighbouring cells and trigger the transcriptional regulation of genes involved in the antiviral state. Studies in mammals have demonstrated that there are several hundred such IFN-regulated genes (IRGs). Because the vast majority are up-regulated they are overwhelmingly referred to as IFN-stimulated genes (ISGs) so, hereafter, they will be referred to generically as ISGs (or specifically as chicken ISGs, ChISGs), except where the more generic term avoids confusion. Induction of ISGs involves the JAK/STAT signalling pathway: STAT1 is either recruited directly to target promoters for a relatively weak activation or, more commonly, is recruited in a complex called ISGF3 in association with STAT2 and IRF9 [1, 3].

ISGs are the focus of considerable current attention with regard to: (i) their antiviral activity, (ii) an increasing appreciation of the complexity of their regulation and (iii) their targeting by virus-encoded modulators of IFN-induced responses [1, 3, 4]. These studies require comprehensive catalogues of the ISGs, especially where system-wide approaches are undertaken. Even though many key mammalian ISGs have been known for some time, it is with the relatively recent advent of transcriptomic technologies that the full complement has been catalogued (mainly using microarrays [5]; see also Schoggins et al. [6]).

In contrast to the mammalian IFN system our equivalent knowledge of the avian system has lagged behind. Although IFN was discovered in chickens in 1957 [7] the first chicken IFN gene was characterised in 1994 [8] and the key chicken ISG, PKR, was identified in 2004 [9]. The derivation of the chicken genome sequence, first drafted in 2004 [10], did not greatly advance our understanding of chicken ISGs because of the incomplete nature of the *Gallus gallus* genome assembly, even at v4 (Galgal4), which might be partly due to the fact that the chicken karyotype has six pairs of macrochromosomes (but 33 pairs of microchromosomes), and the difficulties in annotating immunity genes, which are some of the most divergent between mammals and birds [11]. However, it has become apparent that key genes of the innate immune system, such as the transcription factors IRF9 and one member of the IRF3/IRF7 dyad [12, 13; unpublished], are absent from avian species, indicative of significant functional differences between them and mammals. Moreover, for reasons that are not understood, the cytosolic pattern recognition receptor, RIG-I, appears to have been lost from chicken as well as other galliformes [13, 14].

To generate a chicken ISG database we have compared data from three transcriptomic technology platforms: (i) the classical 3′-biased GeneChip Chicken Genome Array (32K; Affymetrix, High Wycombe, UK), (ii) the Chicken Gene 1.0 Sense Target (ST) whole transcriptome Array (Affymetrix) and (iii) Illumina (Little Chesterford, UK) RNA-seq. This three-way comparison allowed a high level of cross-validation of data from each technology, beyond what would normally be achieved by qRT-PCR. It also allows subsequent studies, constrained to use any particular technology, to be more broadly compared. We monitored IRG expression in chicken embryo fibroblast (CEF) induced for 6 h with 1000 units recombinant chicken IFN-α (rChIFN1; hereafter routinely referred to as IFN), a time chosen to reflect predominantly primary signalling targets. The expression data for selected genes were also validated by PCR and qRT-PCR. Overlapping data show generally high degrees of concordance in the identity of the IRGs and their relative levels of regulation by IFN, with disparity mainly where multiple microarray probes exist for single genes. The study was presented in a preliminary form as a poster at the International Cytokine and Interferon Society (ICIS) meeting (“Cytokines 2015”; October 11–14, 2015) in Bamberg, Germany [15].

## Materials and methods

### Culture, infection and harvesting of CEF for microarray

Freshly isolated CEF were provided by the former Institute for Animal Health (Compton, UK, now The Pirbright Institute, Pirbright, UK). Cells were seeded in T25 flasks (Greiner Bio One, Kremsmünster, Austria; 5.6 × 10^6^ cells/flask) and cultured overnight in 5.5 mL 199 media (Gibco Thermo Fisher Scientific, Paisley, UK) supplemented with 8% heat-inactivated newborn bovine serum (NBCS; Gibco), 10% tryptose phosphate broth (TPB; Sigma-Aldrich, Gillingham, UK), 2% nystatin (Sigma-Aldrich) and 0.1% penicillin streptomycin (Gibco).

### Treatment with IFN

Recombinant chicken IFN-α (rChIFN1) was prepared as previously reported [16] and was added in culture media to a final concentration of 1000 units/mL. Confluent cells were treated with IFN or mock-treated and incubated for six hours before harvesting. Cells were stored at −80 °C in RNAlater (Sigma-Aldrich) until RNA extraction. The experiment was repeated in triplicate with three different batches of CEF.

### RNA extraction and processing of samples for microarray

Total RNA was extracted from cells using an RNeasy kit (Qiagen, Crawley, UK) according to the manufacturer’s instructions. On-column DNA digestion was performed using RNase-free DNase (Qiagen) to remove contaminating genomic DNA. RNA samples were quantified using a Nanodrop Spectrophotometer (Thermo Fisher Scientific, Paisley, UK) and checked for quality using a 2100 Bioanalyzer (Agilent Technologies, Wokingham, UK). All RNA samples had an RNA integrity number (RIN) ≥9.6.

RNA samples were processed for microarray with the GeneChip^®^ Chicken Genome Array (Affymetrix) using the GeneChip^®^ 3′ IVT Express Kit (Affymetrix) or for microarray with the Chicken Gene 1.0 ST Array (Affymetrix) using the Ambion (Paisley, UK) WT Expression Kit for Affymetrix GeneChip^®^ Whole Transcript (WT) Expression Arrays (Ambion) and the GeneChip WT Terminal Labelling and Controls Kit (Ambion), following the manufacturers’ instructions, as described previously [17].

Total RNA (100 ng) was used as input and quality checks were performed using the 2100 Bioanalyzer at all stages suggested by the manufacturer. RNA samples were processed in two batches of 18 but batch mixing was used at every stage to avoid creating experimental bias. Hybridisation of RNA to chips and scanning of arrays was performed by the Medical Research Council’s Clinical Sciences Centre (CSC) Genomics Laboratory (Hammersmith Hospital, London, UK). RNA was hybridised to GeneChip Chicken Genome Array chips (Affymetrix) in a GeneChip Hybridization Oven (Affymetrix), the chips were stained and washed on a GeneChip Fluidics Station 450 (Affymetrix), and the arrays were scanned in a GeneChip Scanner 3000 7G with autoloader (Affymetrix).

### Validation of microarray data for IFN-responsive genes by quantitative real-time PCR (qRT-PCR)

cDNA was synthesised from RNA samples from untreated and IFN-treated CEF using the QuantiTect^®^ Reverse Transcriptase system (Qiagen) according to the manufacturer’s instructions. The cDNA was used as a template in 25 μL RT-PCR reactions containing: 19.35 μL nuclease-free distilled H_2_O (Gibco), 2.5 μL 10× buffer (Invitrogen) 0.75 μL MgCl_2_ (Invitrogen), 0.2 μL dNTPs (25 mm; Sigma-Aldrich), 0.5 μL each of forward and reverse primers (20 pmol/μL; Invitrogen Thermo Fisher Scientific, Paisley, UK), 0.2 μL Taq DNA polymerase (Invitrogen) and 1 μL template cDNA. Primer sequences are shown in Table 1.

**Table 1:** **Primers used to quantify gene expression in mock or IFN-treated CEF using real-time qRT-PCR**

Gene	Accession number	Forward primer (5′–3′)	Reverse primer (5′–3′)
GAPDH	NM_204305.1	GGCACTGTCAAGGCTGAGAA	TGCATCTGCCCATTTGATGT
IFNβ	NM_001024836.1	CAGTCTCCAGGGATGCACAG	GAGAAGGTGGTGGTGAGAGC
MX1	NM_204609.1	CACACCCAACTGTCAGCGAT	ATGTCCGAAACTCTCTGCGG
IFIT5	XM_421662.4	TGCTTCACCAGCTAGGACTCTGC	TGGCTTTTGCTCTGTCACCACTTTG
ZC3HAV1	NM_001012938.1	TCGGCGCCTCTCTACGCCAT	TCAGTCCACTGGCCGTGGTCA
IRF8	NM_205416.1	ACAAGCAGGGCATCTTCATC	TGTTCCCACTCCAGAAGACC
SOCS1	NM_001137648.1	CTGCTGGATGCCTGCGGCTT	GGGCCCGGTCGCGGTTTTAA
IL15	NM_204571.1	CACTGTAAGTGGTCAGACGTTCTGA	GGTTCCTGGCATTCTATATCCTCGT
RSAD2	XM_426208.4	GGACAAGGACGAGACAGTTCC	TCCCGCCTCCTTAAGCATTG
TRIM25	XM_415653.5	TCAAGAGTCCCACCCTTCCA	AGCAGCTCAATGGACAGCAT
LGP2	HQ845773.1	ATCTCGCGGCATTGTCTTCA	CTGCTGCTCATTCTGGGTCA

qRT-PCR was performed using MESA GREEN qPCR MasterMix Plus for SYBR^®^ Assay I dTTP (Eurogentec, Southampton, UK) according to the manufacturer’s instructions. A final volume of 10 μL per reaction was used, with 1 μL cDNA diluted 1:10 in nuclease-free H_2_O as a template. Primers were used at a final concentration of 300 nM. Primer sequences are shown in Table 1. Reactions were performed on an ABI-7900HT Fast Real-Time PCR System (Applied Biosystems, Warrington, UK) using the following programme: 95 °C for 5 min; 40 cycles of 95 °C for 15 s, 57 °C for 20 s, 72 °C for 20 s; 95 °C for 15 s; and 60 °C for 15 s. Data were analysed using SDS 2.3 and RQ Manager software (Applied Biosystems). Glyceraldehyde 3-phosphate dehydrogenase (GAPDH) was used as a reference gene. All target gene expression levels were calculated relative to GAPDH expression levels and the target gene expression level in −2 h uninfected CEF using the comparative C_T_ method (also referred to as the 2^−ΔΔCT^ method).

### RNA-seq

Triplicate untreated (control) and IFN-treated CEF were processed for transcriptome analysis by RNA-seq. The cell samples used were identical to those used for the microarray analyses. Total RNA was extracted as for microarrays (above) and RNA libraries were prepared for deep sequencing using the TruSeq RNA Sample Preparation Kit (Illumina) according to the manufacturer’s instructions. Total RNA (2.5 μg) was used as an input for each library. A total of six RNA adapter indices were randomly assigned to the 12 samples to allow multiplexing of libraries. At the end of the protocol, libraries were quantified using a Nanodrop Spectrophotometer and checked for quality using a 2100 Bioanalyzer High Sensitivity DNA chip (Agilent Technologies). RNA library qPCR quantification, multiplexing and sequencing was performed by the Medical Research Council’s Clinical Sciences Centre (CSC) Genomics Laboratory, Hammersmith Hospital, London, UK. Libraries were quantified using the KAPA Biosystems (London, UK) library quantification kit (KK4824) on an ABI 7500 FAST qPCR machine (Applied Biosystems). Libraries were then diluted to a 2 nM stock solution, pooled for multiplexing, denatured and diluted to a final molarity of 20 pM. Libraries were loaded on to the flow cell (8–16 pM per lane) for clustering and cluster generation was performed by the Illumina cBot using version 3 kits. Sequencing of the flow cell was then carried out on the Illumina HiSeq 2000 using the version 3 kits. Data were processed using RTA version 1.12.4.2, with default filter and quality settings. The reads were demultiplexed (allowing no mismatches in the index sequence) with CASAVA 1.8.1.

### Bioinformatic analysis

Microarray data were processed using workflows in GENESPRING™ (Agilent) and PARTEK™ (Partek Inc., St Louis, MO, USA) commercial software suites.

Data (.CEL files) were analysed and statistically filtered using either Partek Genomic Suite 6.6 (Partek GS) or Genespring version 7.2 (Agilent Technologies) software. Input files were normalized with either GCRMA or Genespring algorithms for gene array on core metaprobesets. A one-way ANOVA was performed using either software across all samples. Statistically significant genes were identified using mixed model analysis of variance with a false discovery rate (Benjamini–Hochberg test) of *P* < 0.05. Fold-change values <±3.0 were removed.

RNA-seq data were imported into CLC bio’s Genomics Workbench (CLC Bio, Aarhus, Denmark; now Qiagen), quality-controlled and thereafter processed using that package (versions 6 and 7).

After quality control, the reads were subjected to quality trimming then mapped against ENSEMBL Galgal4 annotated genes (release 75 [18]) for quantitative analysis of expression. Fold change and False Discovery Rates (FDR) were calculated using Kal’s Z test [19], with pooled data, or Baggerly’s test [20], using separate triplicates.

## Results

Initially, we used the 32K GeneChip^®^ Chicken Genome Array (Affymetrix) because, as well as displaying probes for 32 773 chicken transcripts, it displays probes for 684 transcripts from 17 different viral pathogens of chickens, which offers advantages to those studying virus infections in a chicken background. Subsequently, we used the more refined Chicken Gene 1.0 ST Array (Affymetrix) because it offers a higher probe density against 18 214 chicken genes and should allow detection of transcript isoforms, including non-polyadenylated and alternatively polyadenylated, though it does not include probes for viral genes.

Separate weekly batches of CEF, produced from pools of eggs from the same flock (Rhode Island Red) held in SPF-like conditions at the former Compton Laboratory of the Institute for Animal Health (now The Pirbright Laboratory) served as biological replicates. Principal component analysis of the microarray data (data not shown) indicated limited variation between batches so, thereafter, biological triplicates were used routinely.

IRGs were identified from expression analysis data determined using the 32K GeneChip following IFN treatment (1000 units, 6 h) of CEF. After quantile normalization, significant hits were identified with GENESPRING using an unpaired T test with asymptotic p-value computation and Benjamini–Hochberg multiple testing correction to generate false discovery rates (FDR). A matrix of FDR (from <0.001 to 1) plotted against fold change (FC; from 1.0 to >3) is shown in Table 2. A relatively conservative FDR of <0.01 returned 250 differentially expressed probesets. Overlaying this with a value for FC for which changes in expression might reasonably be expected to be readily and reliably assayed using other technologies, namely >3, reduced the number of selected, significant probesets to a manageable 181 (180 up, 1 down). These settings were therefore chosen for further analysis. For 23 of these probe sets, no currently recognised genes were automatically assigned. Of the remaining 158 probe sets, 29 were assigned to genes recognised in duplicate by other probe sets. Consequently 129 recognised genes were identified as differentially expressed (the down-regulated transcript was not, at that time, assigned to a recognised gene).

**Table 2:** **Matrix showing significant hits from microarray (Affymetrix 32K GeneChip Chicken Genome Array) analysis of chicken embryo fibroblasts (CEF) treated with recombinant chicken IFN1 (1000** **units, 6** **h)**

Fold change	FDR					
	All	<0.05	<0.02	<0.01	<0.005	<0.001
All	38 285	945	414	250	150	24
>1.1	17 636	942	414	250	150	24
>1.5	1965	676	363	232	146	23
>2.0	677	458	296	206	135	22
>3.0	354	306	235	181	123	22
Expected by chance		47	8	2	0	0

Numbers of significant genes are indicated for fold change in expression from >1.1 to >3 and from false discovery rate (FDR) <0.001 to <0.05 (unpaired T test with asymptotic *p* value computation and Benjamini–Hochberg multiple testing correction).

With the Chicken Gene 1.0 ST Array, 157 probe sets demonstrated differential expression (156 up, 1 down) at the same settings (FC > 3, FDR < 0.01). Amongst these, there were five duplicated probe sets and 27 that were not automatically assigned to recognised genes therefore 125 recognised genes were uniquely identified as differentially regulated.

Illumina RNA-seq yielded a total of 170 million reads (100 bases; paired) for the mock-treated CEF triplicate samples and 167 million for the IFN-treated samples. Upon quality trimming and mapping to ENSEMBL Galgal4 annotated genes (release 75), using CLC Bio’s Genomic Workbench, 138 recognised genes were identified as differentially regulated (137 up, 1 down) using Kal’s proportion-based Z test [19; as implemented in the CLC Bio package] at the same settings (FC > 3, FDR < 0.01). Kal’s is performed on the pooled reads from IFN-treated and untreated samples. It is perhaps, therefore, more widely applicable; it also returned a number of IRGs comparable to those returned by the microarrays. Triplicate-based analysis using Baggerly’s proportion-based Beta-binomial test [20; as implemented in the CLC Bio package] at the same settings (FC > 3, FDR < 0.01) returned an additional 37 up-regulated genes.

**Figure 1:**
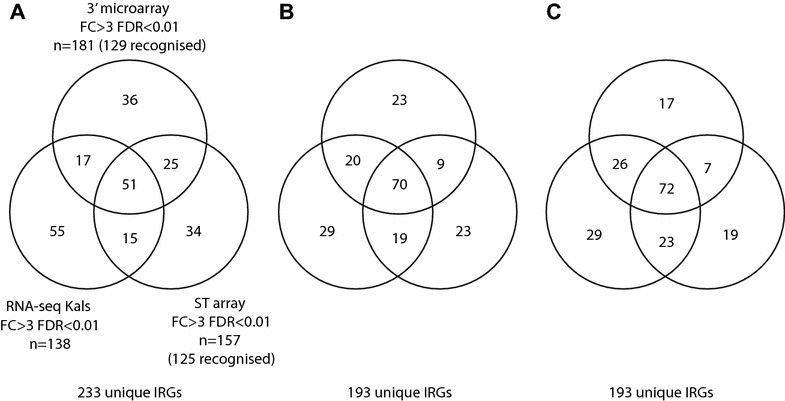
**Correlation of ISGs identified as significant by RNA-seq and microarray.** Venn diagrams showing correlation of significant ISGs (FC ≥ 3; FDR ≤ 0.01, unless stated otherwise) for: (i) Illumina 100b paired-end RNA-seq, (ii) Affymetrix 32K GeneChip Chicken Genome Array and (iii) Chicken Gene 1.0 ST Array, following induction of CEF for 6 h with 1000 units of rChIFN1. (**A**) Total hits (“*n* =”) shown for each technology; those corresponding to *Galgal4* assembly Gene IDs are shown in brackets (“recognised”)—RNA-seq hits all represent *Galgal4* mapped genes. (**B**) Hits from array technologies were manually curated to maximise numbers of corresponding genes. (**C**) Curated array hits shown in (**B**) that are present amongst RNA-seq hits, but at lower levels of significance, were transferred to the respective RNA-seq-overlapping sectors. Total genes are shown for (**A**–**C**).

Comparison of the complete raw gene lists from the three technologies using the most compatible identifier (essentially the Gene Symbol) with an online Venn Diagram tool (Venn Diagram Generator; [21]) demonstrated that 233 recognised genes were identified as differentially regulated. Of these, 51 were identified in common by all three technologies and a further 57 were identified by two out of three technologies, meaning that 108 were identified by at least two technologies. A total of 125 were therefore each identified only by individual technologies (Figure 1A).

**Figure 2:**
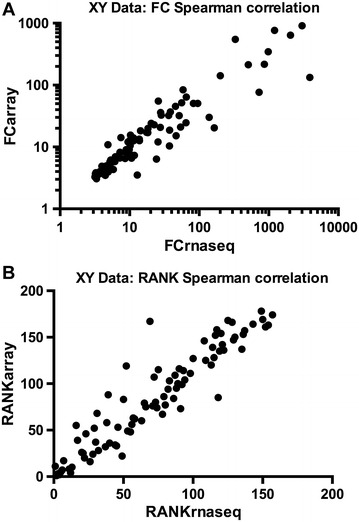
**Comparison of expression level and rank of significant ISGs identified by RNA-seq and microarrays.** Spearman correlation plots for significant ISGs from: (i) Illumina 100b paired-end RNA-seq and (ii) Affymetrix 32K GeneChip Chicken Genome Array, following induction of CEF for 6 h with 1000 units of rChIFN1, by FC (**A**) and by Rank (**B**).

**Figure 3:**
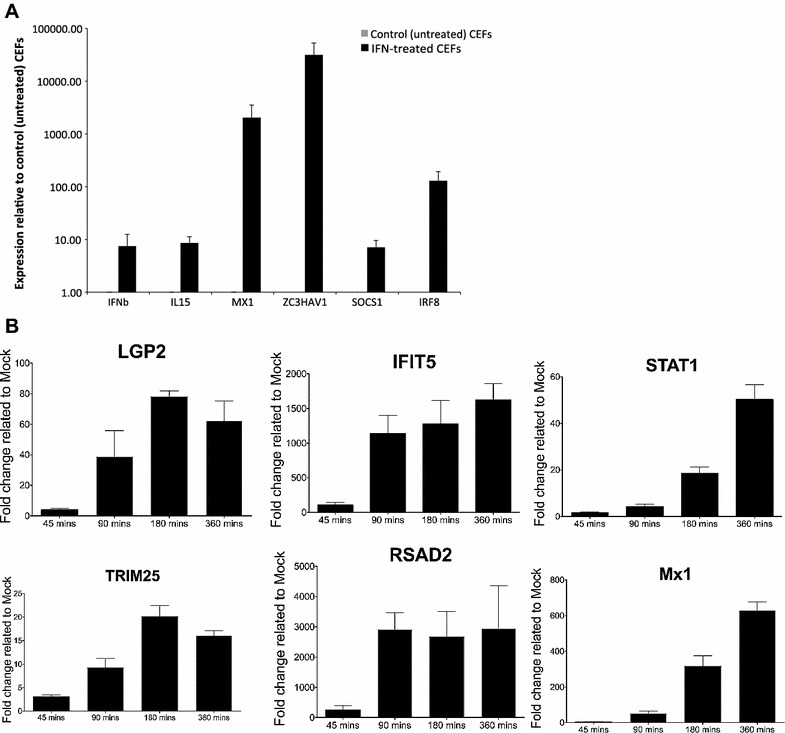
**qRT-PCR validation and kinetic analysis of ISG expression.** (**A**) Validation of RNA-seq and Microarray ISGs by qRT-PCR of CEF treated with recombinant chicken IFN1 (1000 units, 6 h). (**B**) Kinetics of expression of selected ISGs assayed by qRT-PCR following treatment of CEF with recombinant chicken IFN1 (1000 units) for 45, 90, 180 or 360 min. ISGs showing similar kinetic expression profiles are paired vertically.

As well as comparing the identities of the differentially regulated genes, the correlation of expression of the genes identified by the different platforms was examined in terms of both level and rank of FC (Figures 2A and B). For instance, comparing RNA-seq data with the 32K GeneChip data, Spearman correlation values were 0.93 for FC level and rank. Considering the current state of assembly and annotation of the chicken genome, the correlation of ISGs in terms of gene identity as well as the level and rank of induction as indicated by all three technology platforms is reassuring. Nevertheless the platform transcriptomic data were validated for selected genes by RT-PCR (data not shown) and by qRT-PCR (Figure 3A).

A 6 h time point was chosen for microarray and RNA-seq analysis of IFN treatment as it has been widely used and is known to result in significant levels of a broad range of ISGs in mammals, making it suitable for defining the chicken interferome. Use of this single time point does not, however, provide unequivocal insight into mechanistic interpretation of ISG induction; for instance, it does not discriminate between strictly ISRE-dependent induction of ISGs and ISRE-independent induction of ISGs by mechanisms that might include immediate high-level induction of IRF1, which has been observed in mammalian systems [22–24]. Kinetic analysis of the induction of expression of a subset of ISGs was therefore conducted at 45, 90, 180 and 360 min post application of IFN (see Figure 3B). Even among highly-induced ISGs, different temporal profiles were observed, from the rapid accumulation of IFIT5 (1000-fold by 90 min) and RSAD2 (which remain at steady levels to 360 min) to the steadier, sustained accumulation of Mx and the more modestly induced STAT1; with LGP2 and TRIM25 peaking at 180 min. Although differences in mRNA stability and turnover will influence the profiles, this identification of the ISGs will allow detailed analysis of their promoters to investigate elements (and the factors that bind them) that contribute to the complexity of the observed induction patterns.

## Discussion

Of the 51 IRGs initially identified by all three technologies, 47 had mammalian equivalents that are known as ISGs from human or mouse according to the “Interferome” database (v2.01; [25, 26]). Those not listed in Interferome were: EPB41L3, IFI27L2, OLFML1 and TMEM168. Of the 57 IRGs initially identified by two out of the three technologies, 29 have mammalian equivalents known as human or mouse ISGs. Therefore, of the 108 ChISGs identified initially by at least two technologies, 76 were equivalent to known mammalian ISGs. For those ChISGs identified by single technologies, 12 of the 55 identified by RNAseq (L1), 10 of the 36 identified by the 32K Genechip (L2) and 12 of the 34 identified by the ST Array (L3) were listed in Interferome. This added a further 34 candidate ChISGs (a total of 110) with known mammalian ISG equivalents (as recognised by the Interferome database). The majority of ChISGs for which mammalian equivalents cannot be found in the Interferome database (all 4 from the “common” ISGs, 23 of 28 identified by at least two technologies as well as 21 out of 43 for L1, 15 out of 26 for L2 and 13 out of 22 for L3) have gene equivalents in the mammalian genome databases (see Additional files 1 and 2); see also the “ChISG Browser” [Tomlinson, unpublished; 27]). This suggests either that the mammalian equivalents are ISGs but that they are not included as such in Interferome or that they are not ISGs in mammals.

The raw lists were refined by manual “curation”, allowing for synonyms of recognised genes (for instance ISG12-2 versus ISG12(2)) and, after bioinformatic analysis using BLAST, etc., assigning recognised gene identifiers to probe sets that previously lacked them. At the end of this process (Figure 1B; Additional files 1, 2), it was apparent that some (*n* = 12) differentially regulated genes identified by the microarrays were also identified as differentially regulated by RNA-seq but that they fell outside of the strict FC > 3 and FDR < 0.01 parameters, reflecting unsurprising disparity in the sensitivity of the three technologies. Those genes that were expressed down to FC > 2.5 or with an FDR up to < 0.05 were, therefore, also incorporated to produce a final list (Figure 1C; Additional files 1, 2).

It is obvious that this manual curation of the data, to allow for alternative Gene ID nomenclature used by the three technologies and for differences in sensitivity, introduced minor changes to the figures from the automatic comparisons cited above (Figure 1; Additional files 1, 2). Curation, therefore, reduced the number of IRGs from 233 to 193. It also increased the number of differentially expressed genes detected by two out of three technologies from 108 to 118 (compare Figures 1A and B). Relaxing the criteria for detection of differentially regulated genes by RNA-seq (to FC > 2.5 and/or FDR < 0.05) further increased the number of genes detected by all three technologies from 70 to 72 (representing 37%) or by at least two of the technologies from 118 to 128 (66%), leaving 65 genes detected by single technologies (compare Figures 1B and C), with 29 of those detected by RNA-seq alone (using the Kal’s test, at FC > 3.0 and FDR < 0.01; Additional files 1, 2).

Of the 37 additional ISGs identified by RNA-seq as significant (FC > 3 FDR < 0.01) by the more sensitive Baggerly’s test but not by Kal’s (Table 3), two were also identified as significant by Kal’s using the relaxed criteria (FDR < 0.05). Baggerly’s, therefore, identified 35 ISGs additional to those described in the above analyses using RNA-seq (Kal’s analysis) and the microarrays (Table 3).

**Table 3:** **ChISGs identified by RNA-seq using Baggerly’s test but not Kal’s test using standard criteria (FC** **>** **3 or FDR** **<** **0.01)**

Feature ID	Baggerley’s weighted proportions FC	Baggerley’s FDR p value correction	Kal’s relaxed FDR < 0.05^a^
FAM26F	148	0.0000	Yes
THEMIS2	118	0.0004	
ENSGALG00000026152	55	0.0000	
ENSGALG00000005148	54	0.0043	
ENSGALG00000003110	52	0.0050	
IL4I1	26	0.0040	
C1orf168	24	0.0001	
SPIRE2	18	0.0041	
HRH1	17	0.0001	
AZIN2	14	0.0000	
ENSGALG00000029181	14	0.0000	
ENSGALG00000001629	12	0.0001	
B3GNT4	9	0.0000	
GDPD4	8	0.0000	
ATP6V1G3	7	0.0002	
DUSP15	7	0.0002	
IKBKE	7	0.0001	
ANGPTL7	6	0.0000	
ARHGEF28	6	0.0004	
KCNJ5	6	0.0000	
CHRD	5	0.0000	Yes
ENSGALG00000002823	5	0.0005	
ENSGALG00000004772	5	0.0000	
ENSGALG00000006325	5	0.0000	
ENSGALG00000027955	5	0.0000	
FUT10	5	0.0000	
TOR4A	5	0.0001	
C1QTNF1	4	0.0000	
CYBRD1	4	0.0000	
ENSGALG00000000819	4	0.0028	
ENSGALG00000020899	4	0.0000	
ISLR	4	0.0000	
JAM2	4	0.0000	
KIAA0226	4	0.0028	
MALL	4	0.0002	
MAOA	4	0.0000	
RBM43	4	0.0015	

^a^ChISGs significant by Kal’s Z test under relaxed criteria (FDR < 0.05).

### Comparison of technology platforms

Analysis of RNA-seq data depends directly on the extant annotated genome sequence. Perhaps not surprisingly therefore, RNA-seq identified the largest proportion of genes amongst the set of 193 unique IRGs that we compiled (150; 78%). Nevertheless, the microarrays each identified 63% of the genes (122 and 121). Congruence was highest, and almost identical, between RNA-seq and each microarray (98 and 95; 51 ± 1%; all percentages referring to the total of 193 unique IRGs). Between microarrays it fell to 41% (79). For two-way-only comparisons, the distribution of unique genes between the microarrays was symmetrical (42 and 43; 22%). Between RNA-seq and each microarray, unique genes were biased >2-fold towards RNA-seq: 52 (27%) versus 24 (12%) against the Genechip and 55 (28%) versus 26 (13%) against the ST Array.

Clearly in simple terms of numbers of IRGs identified, RNA-seq outperforms the microarrays. This is probably attributable to the historic nature of the array design based on earlier genome assemblies and annotations, with consequent effects on overall coverage (which might disproportionately affect conditionally expressed genes such as those of the innate immune responses). Nevertheless, the ability of microarrays to quantify expression of 50% (about 100) of such a large pool of important genes will often prove sufficient for the experimental objectives where other considerations might affect the choice of technology (see below).

Moving away from actual numbers of genes, it is worth noting that deeper analysis (in the form of validation by alternative approaches) will, by definition, be required to determine which of the genes identified uniquely as IRGs by individual technologies are actually IRGs.

### Identification of ISGs not annotated on the current genome

**Figure 4:**
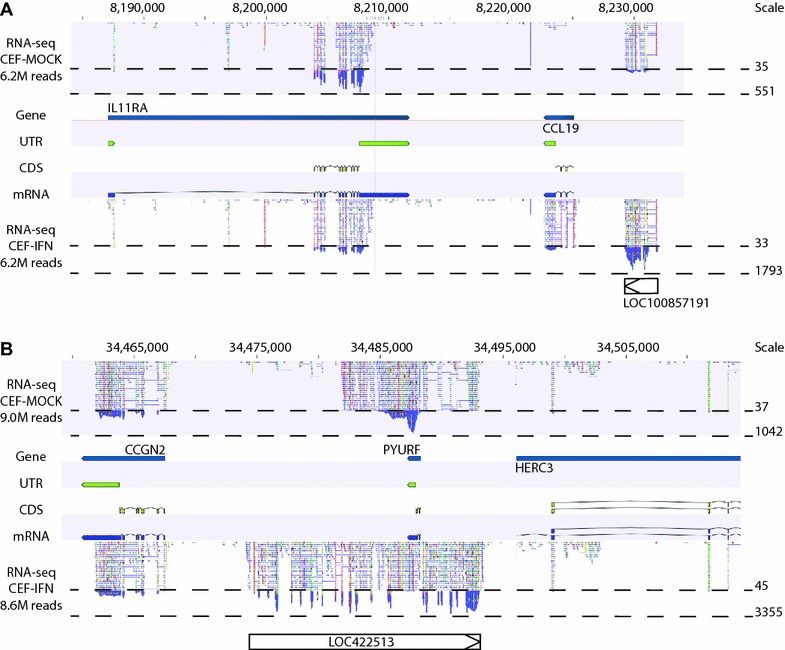
**Gene-level visualisation of RNA-seq reads mapped to the chicken genome.** Annotated CLC Bio Genomic Workbench views of chicken chromosome Z (**A**) and 4 (**B**) showing the loci around homologues of CCL19 (ENSGALG00000028256) (**A**) and PYURF (ENSGALG00000026229) (**B**). Each panel shows tracks for genes (labeled with Galgal4—annotated names), untranslated regions (UTR), coding sequences (CDS) and mRNA transcripts. Locations of unannotated NCBI Galgal5 Refseq genes LOC 1008571891 (**A**) and LOC422513 (**B**) are indicated. RNA-seq reads from untreated and IFN-treated CEF (6 h, 1000 units) are shown mapped to the genome in the uppermost and lowest tracks, respectively, in each panel (totals mapped to the chromosome are indicated to the left of these tracks). The levels of basal and peak RNA-read mappings are shown to the right of the tracks under “Scale”. Comparison of these figures in conjunction with the size of the peaks allows visual estimates of the levels of differential expression for individual exons (which can be compared with the formal numerical analyses). For instance, IL11RA (**A**) as well as CCGN2 and HERC3 (**B**) show no significant regulation by IFN. In contrast, CCL19 and unannotated LOC100857191 in (**A**) show significant upregulation (96-fold—but with an FDR of 0.031 it fell outside the cut-off for Kal’s analysis and, because of its very low basal expression, was not returned by Baggerly’s). In (**B**) PYURF shows 24-fold suppression by IFN but the sequence surrounding PYURF shows 87-fold induction from the right-hand end of the unannotated, antisense LOC422513 and considerably higher upregulation from the left-hand end (due to its lower uninduced levels), consistent with these representing homologues of IFN-inducible human genes HERC6 and HERC5.

Genomic loci for each of the predicted ISGs were visually inspected using Genomic Workbench’s genome browser, displaying tracks showing: gene, transcript, exon and ORF annotations for the current chicken genome build as well as read-mapping for control and IFN-treated reads [27]. On occasions, such inspection revealed the presence of non-annotated, inducibly-transcribed regions, representing exons, whole genes or even gene families. Examples include those previously described at the chicken IFITM locus [28; data not shown], at the HERC locus (described below) or downstream of CCL19 (LOC100857191; “C–C motif chemokine 26-like”; Figure 4). Systematic analysis of these ISGs is outside the scope of this manuscript but the data deposited from this study (European Nucleotide Archive (ENA) study number PRJEB7620 [44]) will facilitate ongoing study and improved annotation. In some cases, although not currently annotated on the ENSEMBL chicken genome, the genes have IDs in NCBI and were identified as ISGs by one of the microarrays. Examples of these include LOC415756, LOC415922 (“guanylate-binding protein 4-like”) and LOC422513 (“hect domain and RLD 4-like”, a member of the HERC family, discussed below).

### Identification of ISGs not present in the current genome

About 10% of the reads from CEFS did not map to the current chicken genome. The unmapped reads combined from the control and IFN-treated samples were assembled into contigs using the *de novo* assembly function of Genomic Workbench. The RNA-seq function of Genomic Workbench was then used to quantitate expression of the contigs in control and IFN-treated samples. One of the most highly-expressed contigs was one which, when analysed by BLAST, proved to represent a homologue of STAT2, which is missing from the current ENSEMBL annotated reference chicken genome assembly (Galgal4; release 84), though at NCBI it has recently been placed as a Refseq gene on chromosome 33 in the new assembly Galgal5 (an annotated form of which has not yet been released and is currently not scheduled for release). The de novo assembled contig sequence was used to derive primers for RT-PCR; characterisation of chicken STAT2 will be reported elsewhere.

### Interferon down-regulated gene expression

The data on differential expression showed an overwhelming over-representation of genes up-regulated by IFN. For each of the technologies, only one gene was detected as down-regulated. Corresponding GeneIDs were PYURF (PIGY upstream reading frame; ENSGALG00000026229) for RNA-seq and PIGY (phosphatidylinositol glycan anchor biosynthesis, class Y; NCBI GeneID: 101748971) for the ST array. The down-regulated 32K GeneChip probe (Gga.8802.1.S1_at), though not mapped to a known gene at the time of initial processing, according to the Affymetrix NetAffx™ Analysis Center [29] is now also assigned as PYURF. In humans, PIGY and PYURF represent different open reading frames on the same spliced transcript of a gene on Hs chromosome 4 located downstream of HERC6 then HERC5. The PYURF/PIGY gene is overlapped on the opposite strand by HERC3, which extends downstream to be followed by FAM13A. Similarly, the chicken PIGY (NCBI) and PYURF (Ensembl) genes map to a locus lying upstream of HERC3 then FAM13A on Gg chromosome 4 (see Figure 4), with HERC-like LOC422513 (“hect domain and RLD 4-like”) starting upstream but spanning and extending downstream of the chicken PYURF. Our RNA-seq data (Figure 4) indicate that this locus is poorly annotated and demonstrates complex regulation of the component genes by IFN. Thus, although the PIGY/PYURF transcript is down-regulated by IFN, as recorded by all three technologies, it appears to be closely flanked upstream and downstream by still unannotated multiple exons that are clearly strongly induced by IFN (Figure 4). Sequences within these upstream and downstream regions (which are represented by the single NCBI Refseq (Galgal5) gene, LOC422513, but appear as though they may represent two separate genes, Figure 4) bear homology with genes of the HERC family, consistent with the fact that HERC5 neighbours the human PIGY/PYURF gene and that HERC3 neighbours the chicken PIGY/PYURF gene. The chicken HERC3 gene shows no evidence of induction by IFN.

Description of the interferon-inducibility of the ChISGs serves as the first step in understanding the regulation of their expression and their role in anti-viral (and potentially broader anti-microbial) activities. There is considerable current interest in the antiviral responses of particular cell types, particularly those of the lymphoid, myeloid and dendritic lineages. However, the definition of a wide variety of these cell types is not so advanced in avian species so we felt it best to produce baseline data for readily available, primary cells, namely chick embryo fibroblasts (CEF) as they are highly responsive to IFN. They also remain important for commercial production of vaccine viruses (including human vaccines) as well as for the routine isolation and diagnosis of avian pathogens.

Given the currently incomplete nature of the chicken genome assembly (even at Galgal5) and of its annotation (as currently available for Galgal4 and even as awaited for Galgal5) it is inevitable that updates will continue to be released but the primary data reported here, and publicly-available, for microarrays and RNA-seq, can always be applied to updated microarray assignments as well as to subsequent genome assemblies and annotations.

All things being equal, RNA-seq would seem to be the method of choice for transcriptomic analysis of chicken IFN responses, particularly given its ability to produce high-resolution quantitative and qualitative data. Moreover the data are readily portable and can be easily mined by others with different research focus. They can also be applied immediately to newly released genome assemblies and annotations (whether global or local), whereas microarray analysis must await the generation of annotation updates for each technology.

However, although the cost of sequencing has fallen, and will probably continue to do so, there remain considerable overheads to handling large data sets from extensive, complicated experiments, especially in terms of computing and data storage capacity, as well as speed of processing and archiving. For such experiments, microarrays continue to offer a tractable approach, capable of quickly quantifying and comparing the expression of the central core of IRGs producing relatively compact data for rapid analysis and easy archiving.

Induction of innate responses with PAMPS will trigger different or broader ranges of responses by virtue of the fact that they will trigger other or more pathways than just the IFN-pathway. For instance we (Giotis et al. unpublished) and others [12] have begun to analyse the responses induced by the dsRNA analogue poly[I:C]. Regulation of ISG expression might affect the innate responses observed in different cell lines or tissues so it will be important to understand the mechanisms involved. Additionally, we have observed suppression of ISG induction in the spontaneously immortalized chicken fibroblast cell line, DF-1 [30], due to their enhanced basal expression of the regulatory ISG, SOCS1 (Giotis et al., unpublished). Identification of the ISGs means that their promoters, enhancers and other regulatory elements can be systematically analysed to help understand the complex kinetics of expression of their expression (Figure 4).

Several studies have investigated changes in host gene expression in response to infection in vivo or in culture with particular avian viruses [31–39]. Although many of these genes will represent innate (and potentially antiviral) host responses, the majority will be involved in the metabolic, cell cycle and ultrastructural changes that the virus has to induce to facilitate replication. Furthermore, it is not unusual for viruses to modulate the expression of signalling molecules key to the antiviral responses or of antiviral effectors themselves. For instance, we have shown that even an attenuated strain of fowlpox virus blocks induction of IFN-β (ChIFN2) and is highly resistant to the antiviral activity induced by IFN [16, 40].

The results of existing and future studies of infection in vivo or in culture with particular avian viruses can now be compared with data presented here for ISG induction by IFN to look for evidence of modulation of ISG expression by viruses, whether that be modulation of individual ISGs, subsets [4] or the complete set. For instance, fowlpox virus blocks essentially all ISG expression but a mutant defective in the fpv012 ankyrin repeat/F-box protein identified by Laidlaw et al. [40] induces modest levels of a subset of the ISGs (Giotis et al., unpublished). Such analyses can be extended to important avian zoonotic viruses and pathogens with huge impact on the global poultry industry. Although this study relates to type I IFN, extensive comparison with the effects of type III IFN could now be conducted, extending on the qRT-PCR comparison made by Masuda et al., who looked at induction of Mx and OAS by IFN-β, IFN-γ and IFN-λ [41].

## Additional files

10.1186/s13567-016-0363-8 **Table of curated ChISGs identified by individual or multiple technologies.** (1) An asterisk indicates a Gene ID not annotated in ENSEMBL. (2) Technologies identifying significant IRGs are listed as ‘1’ RNA-seq (using Kal’s Z test); ‘2’ Affymetrix 32K GeneChip Chicken Genome Array and ‘3’ Chicken Gene 1.0 ST Array’. ChISGs significant by one or both microarrays and RNA-seq using Kal’s Z test under relaxed criteria (FC > 2.5 or FDR < 0.05) are indicated by ‘(1)’. A plus after the technology identifier indicates that IFN-induced RNA-seq read density was observed at the location of the unannotated gene. (3) Indicates whether homologues of the ChISG identified are listed in the Interferome website as induced by interferon in *Homo sapiens* (Hs), *Mus musculus* (Mm), both (Hs/Mm), or neither (***).
10.1186/s13567-016-0363-8 **Detailed information on ChISGs identified by RNA-seq, and microarray technologies (1).** Technologies identifying significant IRGs are listed as “1” RNA-seq (using Kal’s Z test); “2” Affymetrix 32K GeneChip Chicken Genome Array and “3” Chicken Gene 1.0 ST Array’. ChISGs significant by one or both microarrays and RNA-seq using Kal’s Z test under relaxed criteria (FC > 2.5 or FDR < 0.05) are indicated by “(1)”. “+” after the technology identifier indicates that IFN-induced RNA-seq read density was observed at the location of the unannotated gene. (2) Interferome status [45]. (3) Human homologue data (HUGO) [46]. (4) Mouse orthologue data (MGI) [47].

## Abbreviations

IFN: interferon
IRGs: IFN-regulated genes
ISGs: IFN-stimulated genes
CEF: chicken embryo fibroblasts
rChIFN1: recombinant chicken IFN-α
RIN: RNA integrity number
qRT-PCR: quantitative real-time PCR
GAPDH: glyceraldehyde 3-phosphate dehydrogenase
FC: fold change
FDR: false discovery rate
