# Supplementary material for: Chicken interferome: avian interferon-stimulated genes identified by microarray and RNA-seq of primary chick embryo fibroblasts treated with a chicken type I interferon (IFN-α)
Source: Vet Res. 2016 Aug 5;47:75. doi: 10.1186/s13567-016-0363-8 (PMC4974698; doi:10.1186/s13567-016-0363-8)
Supplement: Supplementary file 1 — 10.1186/s13567-016-0363-8 Table of curated ChISGs identified by individual or multiple technologies. (1) An asterisk indicates a Gene ID not annotated in ENSEMBL. (2) Technologies identifying significant IRGs are listed as ‘1’ RNA-seq (using Kal’s Z test); ‘2’ Affymetrix 32K GeneChip Chicken Genome Array and ‘3’ Chicken Gene 1.0 ST Array’. ChISGs significant by one or both microarrays and RNA-seq using Kal’s Z test under relaxed criteria (FC > 2.5 or FDR < 0.05) are indicated by ‘(1)’. A plus after the technology identifier indicates that IFN-induced RNA-seq read density was observed at the location of the unannotated gene. (3) Indicates whether homologues of the ChISG identified are listed in the Interferome website as induced by interferon in Homo sapiens (Hs), Mus musculus (Mm), both (Hs/Mm), or neither (***). [file 13567_2016_363_MOESM1_ESM.doc]

| **Gene ID^1^** | **Synonyms/Alternative name** | **RNA-seq**  **FC** | **RNA-seq FDR** | **Significant by technology^2^**  **(relaxed)** | **IFNome^3^** |
| --- | --- | --- | --- | --- | --- |
| ACKR4 |  | 6.4 | 0.0010 | 1 | Hs |
| ADAR |  | 6.3 | 0.0000 | 123 | Hs/Mm |
| ADC |  | 13.7 | 0.0001 | 1 | Mm |
| ANGPT1 |  | 9.5 | 0.0204 | (1)23 | Hs |
| ANGPTL1 |  | 6.0 | 0.0001 | 123 | Hs |
| ANKFN1 |  | 60.2 | 0.1222 | 2 | *** |
| ARHGAP8 |  | 14.0 | 0.4722 | 3 | Hs |
| ASPA |  | 6.3 | 0.9999 | 3 | Mm |
| ASS1 |  | 6.1 | 0.0000 | 13 | Hs |
| ATP8A1 |  | 20.5 | 0.0005 | 13 | Hs/Mm |
| AVD |  | 3.5 | 0.0000 | 1 | *** |
| B2M |  | 14.9 | 0.0000 | 123 | Hs/Mm |
| B3GNT7 |  | 50.6 | 0.0000 | 12 | *** |
| B4GALNT4 |  | 3.7 | 0.0001 | 1 | *** |
| BATF3 |  | 5.3 | 0.1101 | 23 | Hs/Mm |
| BCAP29 |  | 4.4 | 0.0000 | 12 | Hs |
| BCMO1 |  | 35.9 | 0.0000 | 123 | Hs |
| BF1 |  | 12.1 | 0.0000 | 13 | *** |
| BFAR |  | 3.8 | 0.0100 | 123 | Mm |
| BFIV21 |  | 10.5 | 0.0000 | 1 | *** |
| C1S |  | 3.2 | 0.0000 | 1 | Hs/Mm |
| CALCOCO2 |  | 7.3 | 0.0000 | 123 | Hs |
| CAMKK2 |  | 9.4 | 0.0000 | 123 | Hs/Mm |
| CAPN13 |  | 25.3 | 0.2858 | 3 | *** |
| CASP7 |  | 10.8 | 0.0016 | 123 | Hs/Mm |
| CD274 |  | 11.8 | 0.0000 | 123 | Hs/Mm |
| CDCA7 |  | 4.1 | 0.0001 | 1 | Mm |
| CMPK2 |  | 756.9 | 0.0000 | 123 | Hs/Mm |
| CMTM6 |  | 5.3 | 0.0454 | (1)3 | *** |
| CMTR1 | KIAA0082; FTSJD2 | 13.0 | 0.0000 | 123 | Hs |
| CNP |  | 8.5 | 0.0000 | 12 | Hs/Mm |
| CTSS |  | 9.4 | 0.0000 | 123 | Hs/Mm |
| DDX27 |  | 2.8 | 0.0000 | (1)2 | Mm |
| DDX60 | ENSGALG00000009639; LOC422427 | 61.1 | 0.0000 | 12 | Hs/Mm |
| DHX58 |  | 32.4 | 0.0000 | 13 | Hs/Mm |
| DNAJC13 |  | 3.5 | 0.0007 | 1 | Mm |
| DRAM1 |  | 20.7 | 0.0000 | 123 | Hs/Mm |
| DTX3L |  | 29.0 | 0.0000 | 123 | Hs/Mm |
| EFHD1 |  | 4.7 | 0.0000 | 12 | *** |
| EIF2AK2 |  | 23.7 | 0.0000 | 123 | Hs/Mm |
| EIF4E3 |  | 5.2 | 0.0067 | 13 | Hs |
| ENSGALG00000000720 |  | 61.9 | 0.0000 | 13 | *** |
| ENSGALG00000001398 | PML | 13.1 | 0.0014 | 123 | Hs/Mm |
| ENSGALG00000001478 | PML-like | 16.8 | 0.0000 | 123 | *** |
| ENSGALG00000002102 | LOC415325 | 719.7 | 0.0242 | (1)3 | *** |
| ENSGALG00000003572 |  | 5.6 | 0.0006 | 13 | *** |
| ENSGALG00000006384 | IFIT5 | 2555.7 | 0.0000 | 123 | Hs |
| ENSGALG00000009479 | SAMD9L | 2860.9 | 0.0000 | 123 | Hs/Mm |
| ENSGALG00000011190 |  | 684.7 | 0.0000 | 1 | *** |
| ENSGALG00000011449 |  | 4.0 | 0.0001 | 123 | *** |
| ENSGALG00000012072 | PARP14 | 61.2 | 0.0000 | 123 | Hs/Mm |
| ENSGALG00000013057 | USP18 | 97.1 | 0.0000 | 123 | Hs/Mm |
| ENSGALG00000014659 |  | 3.5 | 0.0000 | 1 | *** |
| ENSGALG00000016556 | GVINP1, GVIN1, VLIG | 554.6 | 0.0000 | 123 | Hs/Mm |
| ENSGALG00000016572 | C3H8orf80 | 52.0 | 0.0002 | 123 | *** |
| ENSGALG00000019141 | LyG1 | 271.7 | 0.0000 | 12 | *** |
| ENSGALG00000019325 |  | 40.5 | 0.0000 | 13 | *** |
| ENSGALG00000023485 |  | 19.7 | 0.0006 | 1 | *** |
| ENSGALG00000023709 | TOR1BL | 72.6 | 0.0000 | 123 | *** |
| ENSGALG00000024348 |  | 6.0 | 0.0012 | 1 | *** |
| ENSGALG00000026422 |  | 155.8 | 0.0000 | 1 | *** |
| ENSGALG00000026970 | IFITM3 | 5.4 | 0.0000 | 12 | Hs/Mm |
| ENSGALG00000027067 | C1QTNF1 | 3.8 | 0.0000 | 1 | Hs only |
| ENSGALG00000028016 |  | 44.5 | 0.0009 | 1 | *** |
| EPB41L3 |  | 5.4 | 0.0000 | 123 | *** |
| EPSTI1 |  | 537.5 | 0.0000 | 123 | Hs/Mm |
| ERAP1 |  | 7.1 | 0.0000 | 123 | Hs/Mm |
| ETV7 |  | 20.7 | 0.9156 | 2 | Hs |
| FAM149A |  | 8.3 | 0.9999 | 23 | *** |
| FANCA |  | 4.9 | 0.9999 | 2 | Hs/Mm |
| FMR1 |  | 3.3 | 0.0002 | 12 | Hs/Mm |
| FNDC3A |  | 3.4 | 0.0013 | 1 | Mm |
| GCH1 |  | 3.0 | 0.0585 | 2 | Hs/Mm |
| GFPT2 |  | 3.0 | 0.0001 | (1)2 | Hs/Mm |
| gga-mir-147_1 |  | 27.5 | 0.0000 | 123 | *** |
| GGT5 |  | 14.5 | 0.0000 | 123 | Hs |
| GKAP1 |  | 5.8 | 0.0001 | 1 | *** |
| GPR137B |  | 2.7 | 0.2120 | 2 | Hs |
| HELB |  | 5.1 | 0.1568 | 3 | Hs |
| HELZ2 |  | 17.6 | 0.0000 | 13 | Hs/Mm |
| IFI27L2 |  | 44.3 | 0.0000 | 123 | *** |
| IFI35 |  | 30.7 | 0.0000 | 123 | Hs/Mm |
| IFIH1 |  | 63.5 | 0.0000 | 123 | Hs/Mm |
| IFITM5 |  | 5.8 | 0.0085 | 1 | Hs/Mm |
| IP3KA | ITPKA | 5.7 | 0.0076 | 13 | Mm |
| IQCK |  | 1.7 | 0.9999 | 2 | Hs |
| IRF-3 |  | 14.9 | 0.0000 | 123 | *** |
| IRF1 |  | 19.5 | 0.0000 | 123 | Hs/Mm |
| IRF10 |  | 14.8 | 0.0002 | 13 | *** |
| IRF8 |  | 5.0 | 0.0000 | 123 | Hs/Mm |
| ISG12(2) |  | 885.6 | 0.0000 | 123 | *** |
| ISLR2 |  | 14.2 | 0.0000 | 13 | *** |
| JPH1 |  | 3.2 | 0.9665 | 2 | *** |
| KAT2A |  | 1.4 | 0.9999 | 2 | *** |
| KIF5C |  | 3.8 | 0.0041 | 12 | Mm |
| KLHL10 |  | 1.4 | 0.9999 | 3 | *** |
| LIPA |  | 3.3 | 0.0000 | 1 | Mm |
| LRP3 |  | 4.8 | 0.3702 | 3 | *** |
| LRRC15 |  | 4.9 | 0.0000 | 1 | *** |
| LY6E | LyG1 | 9.0 | 0.0000 | 12 | Hs/Mm |
| LY75 |  | 7.3 | 0.0000 | 123 | Mm |
| LY96 |  | 34.6 | 0.5142 | 3 | Hs/Mm |
| LYG2 |  | 24.9 | 0.2020 | 23 | *** |
| MAN2A1 |  | 3.8 | 0.0001 | 1 | *** |
| MITD1 |  | 11.5 | 0.0000 | 123 | Hs/Mm |
| MLH1 |  | 3.8 | 0.3399 | 2 | *** |
| MOV10 |  | 66.0 | 0.0000 | 123 | Hs/Mm |
| MX |  | 1225.2 | 0.0000 | 123 | Hs/Mm |
| MXI1 |  | 7.4 | 0.0000 | 123 | Hs |
| MYD88 |  | 4.7 | 0.0000 | 12 | Hs/Mm |
| NAAA |  | 9.1 | 0.0856 | 3 | Mm |
| NBN |  | 4.6 | 0.0005 | 123 | Hs |
| NCOA7 |  | 4.9 | 0.0013 | 12 | Hs/Mm |
| NKTR |  | 3.3 | 0.0076 | 12 | Mm |
| NLRC5 |  | 4.8 | 0.9999 | 3 | Hs/Mm |
| NMI |  | 14.1 | 0.0000 | 123 | Hs/Mm |
| NT5C3B |  | 8.5 | 0.0000 | 123 | *** |
| OAS*A |  | 755.9 | 0.0000 | 123 | Hs |
| OGFR |  | 10.9 | 0.0000 | 123 | Hs/Mm |
| OLFML1 |  | 171.5 | 0.0000 | 123 | *** |
| OSBPL1A |  | 2.3 | 0.9999 | 2 | Hs/Mm |
| OTUD4 |  | 3.2 | 0.0588 | 23 | Hs |
| P2RX7 |  | 19.1 | 0.0130 | (1)3 | Hs/Mm |
| PARP12 |  | 23.1 | 0.0000 | 123 | Hs/Mm |
| PARP9 |  | 19.4 | 0.0000 | 123 | Hs/Mm |
| PATL2 |  | 35.0 | 0.0074 | 1 | *** |
| PBEF1 |  | 3.7 | 0.0000 | 1 | *** |
| PFKP |  | 4.5 | 0.0000 | 1 | Hs/Mm |
| PHF11 |  | 10.0 | 0.0000 | 123 | Hs |
| PIGA |  | 6.3 | 0.0058 | 123 | Mm |
| PLA2G6 |  | 4.5 | 0.1065 | 23 | *** |
| PLAC8 |  | 9.7 | 0.9999 | 3 | Hs/Mm |
| PPM1J |  | 23.5 | 0.0094 | 12 | *** |
| PRMT8 |  | 5.5 | 0.0094 | 123 | Hs |
| PTGES |  | 6.0 | 0.0000 | 13 | Hs/Mm |
| PXK |  | 8.8 | 0.0000 | 123 | Hs/Mm |
| PYURF |  | -6.4 | 0.0002 | 123 | *** |
| RASSF5 |  | 13.0 | 0.9999 | 2 | *** |
| RFC1 |  | 2.8 | 0.0054 | (1)2 | *** |
| RFFL |  | 4.5 | 0.0172 | (1)2 | *** |
| RHOC |  | 3.7 | 0.0000 | 1 | Hs/Mm |
| RNF19B |  | 4.9 | 0.1173 | 2 | Hs/Mm |
| RNF213 |  | 201.0 | 0.0000 | 13 | Hs/Mm |
| RP9 |  | 6.6 | 0.0000 | 13 | Hs |
| RPA3 |  | 3.3 | 0.0014 | 12 | *** |
| RSAD2 |  | 1505.1 | 0.0000 | 123 | Hs/Mm |
| SAMHD1 |  | 4.7 | 0.0003 | 123 | Hs/Mm |
| SLBP |  | 6.4 | 0.0138 | (1)23 | Mm |
| SLC2A6 |  | 24.6 | 0.0035 | 13 | Hs/Mm |
| SLC35F1 |  | 3.1 | 0.9999 | 2 | *** |
| SMC6 |  | 4.5 | 0.0893 | 23 | *** |
| SMCHD1 |  | 5.4 | 0.0000 | 123 | Hs/Mm |
| SNX10 |  | 10.1 | 0.0000 | 123 | Hs/Mm |
| SOCS1 |  | 18.7 | 0.0000 | 123 | Hs/Mm |
| SPOCK1 |  | 4.5 | 0.0094 | 12 | Hs |
| STAT1 |  | 37.1 | 0.0000 | 123 | Hs/Mm |
| STEAP3 |  | 8.3 | 0.7277 | 3 | *** |
| STOML1 |  | 12.2 | 0.0000 | 123 | Hs |
| TAP1 |  | 27.9 | 0.0000 | 12 | Hs/Mm |
| TAP2 |  | 19.6 | 0.0000 | 12 | Hs/Mm |
| TAPBPL |  | 8.7 | 0.8881 | 3 | Hs/Mm |
| TGM2 |  | 3.9 | 0.0000 | 12 | Hs/Mm |
| TJP2 |  | 5.6 | 0.0000 | 123 | Hs/Mm |
| TLR3 |  | 141.0 | 0.0000 | 123 | Hs/Mm |
| TMEM168 |  | 4.1 | 0.0046 | 123 | *** |
| TMEM173 | STING | 51.6 | 0.0320 | (1)3 | Hs/Mm |
| TMEM52 |  | 4.0 | 0.9999 | 3 | *** |
| TNFRSF18 |  | 13.4 | 0.9999 | 3 | Mm |
| TNFRSF4 |  | 24.8 | 0.0003 | 13 | *** |
| TOR1AIP2 |  | 3.8 | 0.0058 | 1 | Mm |
| TOR4A | C9orf167 |  |  | 2 | Mm |
| TPN |  | 6.9 | 0.0000 | 1 | Hs/Mm |
| TRAF-5 |  | 10.1 | 1.0000 | 23 | Hs |
| TRAFD1 |  | 12.1 | 0.0000 | 123 | Hs/Mm |
| TRANK1 |  | 38.6 | 0.0000 | 123 | Hs |
| TRIM25 |  | 13.5 | 0.0000 | 123 | Hs/Mm |
| TTC3 |  | 3.2 | 0.0138 | (1)2 | Hs/Mm |
| UBE2L6 |  | 14.6 | 0.0000 | 1 | Hs/Mm |
| USP25 |  | 3.5 | 0.1325 | 2 | Hs/Mm |
| VAT1 |  | 5.1 | 0.0000 | 12 | *** |
| VCAM1 |  | 60.2 | 0.0000 | 13 | Hs/Mm |
| WDFY2 |  | 4.2 | 0.0001 | 1 | *** |
| ZC3HAV1 |  | 50.1 | 0.0000 | 123 | Hs/Mm |
| ZCCHC11 |  | 3.2 | 0.0314 | (1)2 | Mm |
| ZDHHC4 |  | 10.2 | 0.0004 | 13 | *** |
| ZNFX1 |  | 53.7 | 0.0000 | 123 | Hs/Mm |
| * LOC415922 | GBP4L, GBP7 |  |  | 3+ | Mm |
| * LOC422513 | hect domain RLD 4-like, HERC5 |  |  | 3+ | Hs |
| * PDCD1LG2 |  |  |  | 3 | Hs |
| * PRLHR2 |  |  |  | 3 | *** |
| * TMEM140 |  |  |  | 3 | Hs/Mm |
| * LOC415756 | C19orf12 |  |  | 2+ | Hs |
| * LOC419112 | ENSGALG00000026622, TMEM110L |  |  | 2 | Hs/Mm |
